# Abortion as an Essential Health Service in Latin America During the COVID-19 Pandemic

**DOI:** 10.3389/fgwh.2022.898754

**Published:** 2022-08-01

**Authors:** Agustina Ramón Michel, Sonia Ariza Navarrete, Susana Chávez

**Affiliations:** ^1^Centro de Estudios de Estado y Sociedad, Health Area, Latin American Consortium Against Unsafe Abortion, Palermo University, Buenos Aires, Argentina; ^2^Centro de Estudios de Estado y Sociedad, Latin American Consortium Against Unsafe Abortion, Lisboa, Portugal; ^3^Latin American Consortium against Unsafe Abortion, Centro de Promoción y Defensa de los Derechos Sexuales y Reproductivos, Lisboa, Portugal

**Keywords:** abortion, Latin America, COVID-19, essential health service, green wave

## Abstract

**Background:**

The rapid increase in demand for health services as a result of the COVID-19 outbreak has created significant challenges for health systems. National and international health authorities have declared reproductive health services as essential, particularly those related to prevention, care during pregnancy, delivery and postpartum, as well as abortion services. This research was conducted by a regional team in cooperation with nine local organizations that are members of the Latin American Consortium against Unsafe Abortion (*Consorcio Latinoamericano Contra el Aborto Inseguro*, CLACAI).

**Objectives:**

Our research aimed to examine the provision of reproductive healthcare services, with a focus on abortion, in nine countries during the first few months of the pandemic (March to September 2020).

**Methods:**

Our research design developed a set of quantitative and qualitative indicators to monitor the availability and accessibility of abortion services during the COVID-19 pandemic. Researchers collected the quantitative data by reviewing regulations and other documents, government and civil society reports, and official statistics; the qualitative data was acquired through interviews with key actors, and non-representative surveys completed by healthcare professional and end users of reproductive services.

**Results:**

Although six of the nine countries we researched deemed reproductive health services essential, only two of these six countries considered abortion services to be essential, and all nine countries reported difficulties in accessing abortion services. Restrictive abortion laws remained in place in the majority of countries (seven), and as a result access to abortion services became even more limited than it had been before the pandemic. At the same time, good practices to facilitate access to abortion services in healthcare facilities, updated regulatory frameworks, and collaboration between civil society and government agencies were identified and should continue to be promoted even after the pandemic crisis has subsided.

**Conclusions:**

The pandemic catalyzed what was already happening in each country, and as such abortion services have become more accessible in countries like Argentina, where the so-called green wave has been generating social, legal and policy changes, whereas in countries such as Ecuador, where abortion is legally restricted and opposed to by the government, access to safe abortion became even more difficult than it was before the pandemic. However, the general trend has been a lack of adequate adaptation in order to guarantee quality in abortion care. That said, there have also been some interesting and positive service provision initiatives, such as telemedicine, implemented in at least two countries, which, if maintained long-term, could improve access to safe abortion.

## Introduction

During the COVID-19 pandemic, limitations in access to abortion services around the world were exposed. While threats to overturn legality or the continued enforcement of unreasonable conditions for abortion, which consequently restrict safe access, are not new nor limited to the pandemic, they became more worrying during it. However, some positive changes, such as the use of tele-abortion care—which was resisted in the past—have taken place in some countries.

Latin America and Africa were the most vulnerable regions to COVID-19 due to their high levels of labor informality, poverty, inequality, and their fragile healthcare and social protection systems (1). Since the beginning of the pandemic through January 2021, more than two million people died (2). Although vaccines are now helping to control the number of fatalities, infections persist, and healthcare systems are still having trouble handling the burden of disease caused by the pandemic, leaving many healthcare needs unattended.

Even though most countries have strived to improve access to healthcare services, many of them have not been able to get back to pre-pandemic healthcare coverage levels, producing what is now being called a prolonged health crisis (3).

Within this context, the legal status of abortion plays a relevant role as it frames policy decisions and also influences the medical and social perceptions of this health intervention. While there have been significant advances in terms of legalizing abortion in the last two decades in Uruguay, Argentina, Colombia and several Mexican States, in most countries it is still criminalized. This accounts for the unsatisfactory maternal mortality ratio (MMR), which is far below the target set by the Sustainable Development Goals to lower MMR by 70% compared to 1990 (4).

This difficult situation was exacerbated during the pandemic. Most countries adopted unprecedented measures (including mobility restrictions, lockdowns, and changes to or closures of healthcare services) during the pandemic. These measures led to limitations in healthcare services, including abortion care (5), without taking into account that sexual and reproductive health services are considered essential (cannot be suspended) by many health global governance authorities, including the World Health Organization (WHO).

In this article, we present the results of our research on the provision of abortion as an essential reproductive service. The study was based on nine Latin American countries and carried out between March and September 2020 by nine local NGOs and research organizations, under the regional coordination of the Latin American Consortium against Unsafe Abortion (CLACAI) (Table 1). We developed a set of indicators that allowed us to understand the landscape of reproductive health services and abortion care by analyzing quantitative and qualitative data during the COVID-19 outbreak.

**Table 1:** Organizations involved in the study.

**Country**	**Organization**	**Web page**
Argentina	Equipo Latinoamericano de Justicia y Género –ELA	https://www.ela.org.ar/a/APP187
Bolivia	Católicas por el Derecho a Decidir -CCD	https://catolicasbolivia.org/
Brasil	Instituto de Bioética, Direitos Humanos e Gênero – Anis.	http://anis.org.br/
Chile	Corporación Miles	https://mileschile.cl/en/
Colombia	Centro de Derechos Reproductivos -CDR	https://reproductiverights.org/our-regions/latin-america-caribbean/
Ecuador	Surkuna	https://surkuna.org/
El Salvador	Agrupación ciudadana por la despenalización del aborto	https://agrupacionciudadana.org/
Perú	Promsex	https://promsex.org/
Uruguay	Mujer y Salud –MYSU	https://www.mysu.org.uy/web/
Regional	Consorcio Latinoamericano contra el aborto inseguro- CLACAI	https://clacai.org/

*Source. Prepared by authors*.

We discuss specific abortion-related public policies undertaken during the crisis. There is no doubt that Latin America is experiencing a wave of changes in relation to abortion—which includes original ways to provide the service—that has continued to gain traction during the pandemic, although not in a uniform fashion, as there are variations from country to country.

This study is of interest given the limited body of work on reproductive health during the pandemic in Latin America (5). This article hopes to contribute to the knowledge on barriers and innovative solutions to accessing safe abortion services during the pandemic, and the adaptations and changes that should remain in place in the years in the post-pandemic world.

## Materials and Methods

The wider study focuses on the availability and accessibility of essential reproductive healthcare services in the first year of the COVID-19 pandemic in Latin America (March to September 2020) (6, 7): prenatal care, childbirth, and postpartum care; counseling and provision of contraceptive methods; safe abortion services. In this paper we share findings related to abortion services (Tables 2–4).

**Table 2:** Structural indicators^*^.

**Structural indicators**			
**N**.	**Essential service**	**Type of indicator**	**Indicator**
1.	All	Availability	Recognition of reproductive healthcare services as essential and urgent
1.1	All	Availability	Restrictions for reproductive healthcare services
2.	All	Availability	Adoption of telehealth to provide sexual and reproductive healthcare services
2.1	Abortion/voluntary termination of pregnancy (VPT)		Adoption of telehealth to provide VPT
2.2	Reproductive Counseling		Adoption of telehealth to provide counseling
3.	All	Accessibility	Adoption of outpatient reproductive healthcare at all levels of care in a healthcare system
3.1	VPT		Adoption of outpatient VPT at all levels of care in a healthcare system
4.	All	Accessibility	Removal of unnecessary prerequisites to access reproductive healthcare services.
4.1	VPT		Removal of waiting periods and other unnecessary prerequisites to access VPT
5.	All	Availability	Availability of supplies of equal of better quality than the previous year
5.1	VPT		Availability of supplies (medication and MVA) in sufficient amounts

*Source. Prepared by authors*.

^*^Measures the acceptance and commitment of the States to uphold human rights by adopting legislative, political, and regulatory frameworks, policies, and mechanisms to enforce respect, protection, and compliance with these rights. In this case, we will focus on the implementation of the recommendations proposed in the document in a broader sense to guarantee reproductive health during the COVID-19 pandemic.

**Table 3:** Process indicators^*^.

**Process indicators**			
**N**.	**Essential services**	**Type of indicator**	**Indicator**
6.	VPT	Availability	Reducing, suspending, or restricting provision of VPT services
7.		Accessibility	Implementation of accessibility adaptations in the provision of RH
7.1	VPT		Provision of VPT via telemedicine (yes/no)
7.2	VPT		Provision of outpatient VPT at all levels of care
7.3	VPT		Provision of VPT medications on the first appointment
7.4	VPT		Flexibility with the prerequisites to purchase VPT medication
7.5	VPT		Adaptations for provision of services in rural or hard to access areas
7.6	VPT	Acceptability	Adaptations for provision of VPT to G&T, DPs, indigenous populations, among others. Number of VPT appointments by age, DPs, indigenous populations.
8.	All	Accessibility	Dissemination of information about RH services during the pandemic
9.	All	Quality	Training of healthcare professionals to provide RH services via telemedicine Number of trained professionals
9.1	All	Quality	Dissemination of information to healthcare professionals about adaptations to RH services due to COVID
10.	All	Accessibility	Budget allocation for RH in 2020 Budget allocation for RH in 2019

*Source. Prepared by authors*.

^*^Measures the continued efforts of the States to transform the legal and political commitments toward the desired results by designing, implementing, and monitoring programs for the progressive realization of human rights. In this case, we measure implementations of identified and recommended actions and corresponding resource allocations.

**Table 4:** Outcome indicators^*^.

**Outcome indicators**	
**N**.	**Indicator**
11.	Global fertility rate Fertility rate by age
12.	Maternal mortality 2020 Maternal mortality 2019
13.	Births by age of the pregnant woman 2020 Births by age of the pregnant woman 2019
14.	Forced pregnancies 2020 Forced pregnancies 2019
16.	Abortion complications 2020 Abortion complications 2019
17.	Number of VPTs 2020 Number of VPTs 2019

*Source. Prepared by authors*.

^*^Compiles the outcomes of structural and process initiatives intended to respect, protect, and enforce human rights. We collate the ramifications of structural and process efforts meant to respect, protect, and comply with reproductive rights that we identified as essential.

The overall design of this study includes a set of 18 specific indicators to monitor the availability and accessibility of reproductive healthcare services during the COVID-19 pandemic, including structural, process, and outcome indicators. These indicators allowed us to measure the adoption, implementation, and initial consequences of reproductive health policies. It also enabled us to observe variations during the pandemic, using the previous year as a baseline.

We took the following indicators into account when developing our own: 17 UNFPA sexual and reproductive health indicators (8); SDG reproductive health indicators (9); indicators from the report of the United Nations Commission on Human Rights Special Rapporteur on the right of everyone to the enjoyment of the highest attainable standard of physical and mental health (10); WHO/PAHO 2019 core health indicators (11); 2015 OAS progress indicators for measuring rights under the protocol of San Salvador (12).

Measurements include quantitative and qualitative data. Qualitative data allows us to analyze context and better understand the quantitative data—or the lack of it—as in some cases official quantitative data was not available. During the data collection process, researchers requested information from official sources (public health authorities), interviewed key informants (healthcare professionals, health authorities, and end users), and carried out online non-representative qualitative surveys for healthcare professionals and end users using free online platforms (SurveyMonkey and Facebook). Data collection methods were adapted to each local context depending on the possibility of accessing the available information in real time during the first year of the COVID-19 pandemic.

Data collection was carried out by nine member organizations of the Latin American Consortium against Unsafe Abortion (CLACAI) (Table 1) under the *Reproductive Health is Vital* initiative. Each country gathered information using the proposed indicators and drafted a national report (13). A regional team including the authors of this article systematized the findings to provide a regional overview of access to reproductive healthcare services. Researchers collected data between March and September 2020 using available resources in light of the urgency to issue recommendations to avoid a serious health crisis such as those experienced in the past, such as during the Ebola epidemic. This publication presents results related to safe abortion access during the pandemic.

This study did not require ethical committee approval as it does not qualify as human subjects' research.

## Findings

This section features some of the findings that we considered most relevant to the availability and accessibility of formal safe abortion services in nine countries in Latin America: Argentina, Bolivia, Brazil, Chile, Colombia, Ecuador, El Salvador, Peru, and Uruguay.

We divided this section into four parts. *Abortion's Legal Status During the COVID-19 Pandemic* gives an overview of the status of abortion regulations in the countries studied, highlighting any regulatory changes during the pandemic. *Access to Safe Abortion During the Pandemic* sets out several core indicators on availability of and access to abortion services, pointing out any changes to the 2019 baseline. *Barriers to Accessing Abortion* underscores the main obstacles identified in access to this practice during the first year of the pandemic. Finally, *Access to Abortion During the Pandemic: Best Practices* introduces the facilitators and the innovative strategies reported in the different countries studied to guarantee the provision of safe abortions during the period in question.

### Abortion's Legal Status During COVID-19 Pandemic

Across Latin America we see a wide range of regulations: in some countries, there is an absolute ban on abortion or highly restrictive legal grounds are in place (e.g., abortion only legal in cases of rape), and in others on-demand abortions until 12–14 weeks of pregnancy are legal. Table 5 shows the differences that existed in the region in the countries participating in the study prior to the COVID-19 pandemic.

**Table 5:** Abortion regulations during the pandemic.

**Country**	**Abortion regulations**				
	**On-demand abortion**		**On health grounds**	**On rape grounds**	**On fetal impairment grounds**
	**12 weeks**	**14 weeks**			
Argentina		X	X	X	
Bolivia			X	X	
Brazil				X	X
Chile			X	X	
Colombia*			X	X	X
Ecuador			X	X	
Peru			X		
Salvador					
Uruguay	X		X	X	

*Source. Prepared by authors*.

^*^Colombia recently modified its regulations through a sentence from the Constitutional Court on February 21st, 2022. The ruling allows on-demand abortion for the first 24 weeks, and after that time, the justifications on the table apply. We conducted this study when the applicable regulations were as stated on the table.

Moreover, regulatory changes on abortion were implemented across the region during the pandemic, as shown in Table 6. From the recognition of reproductive health services as urgent and essential; to the issuance of specific guidelines for the provision of abortion during the pandemic; along with flexibility in the prerequisites of access to abortion services considering pandemic restrictions and security measures, on the one hand, aimed to broaden access. And, on the other hand, modifications tightening restrictive requirements impeding proper access, such as the mandatory complaint in cases of rape, or residence in the health service jurisdiction, along with some COVID-19 bio-security measures that we considered excluding or prohibitive to access abortion services. All these later requirements cannot be justified clinically nor in terms of the safety of the practice.

**Table 6:** Regulatory changes on abortion during the pandemic^*^.

**Country**	**Regulatory changes during the pandemic**				
	**Recognition as essential service**	**Specific guidelines for abortion**	**Flexibility with prerequisites**	**Tightening prerequisites not associated to the pandemic**	**COVID-19 exclusionary prerequisites**
Argentina	X		X		
Bolivia	/	X			X
Brazil	/			X	X
Chile		X ^⊛^			X
Colombia	X	X	/		
Ecuador	/				X
Peru	/				X
Salvador					
Uruguay	X				X

*Source. Prepared by authors with data from the “Reproductive Health is Vital” monitoring*.

^*^Modifications enacted from march to October 2020.

*The / stands for partially, because RH is recognized as essential, but it does not expressly include abortion*.

^⊛^*Restrictive Guideline*.

Not all countries included legal abortion within the reproductive health provisions enacted to guarantee access during COVID-19 pandemic. For example, in Ecuador, Peru, and Bolivia some of the reproductive health services, such as pregnancy care and childbirth, were considered essential but safe abortion was not. Chile and El Salvador did not recognize reproductive health or related practices as essential during the pandemic at all. As seen in Table 6, abortion was only deemed an essential service in Argentina, Colombia and Uruguay.

Regulatory adaptations for abortion services implemented during the pandemic favored access to safe abortion in some countries, but in others prerequisites unrelated to COVID-19 were tightened, making access more difficult, as seen in Table 6. The latter is what happened in Brazil, where Ordinance of the Ministry of Health No. 2,282 (27th August, 2020. Procedure for Justification and Authorization of Interruption of Pregnancy, within the scope of the Unified Health System-SUS), imposed, among other prerequisites, the obligation of reporting the rape to the police in order to carry out an abortion in the event of rape. As recognized by many human rights organizations, imposing prerequisites that are not clinically necessary for safety creates unjustified delays in access, and puts the health and other rights of women and other pregnant persons at risk (14–18). We will come back to Brazil's case later.

Most of the countries studied did not relax the legal requirements to access abortion during the pandemic, as seen in Table 6. Argentina and Colombia were the exceptions. In Argentina, the National Ministry of Health and many provincial jurisdictions adapted protocols to promote, among others, the use of telemedicine for the provision of legal termination of pregnancy, hand out, or prescribe the medication at the first appointment, and outpatient abortions at the primary care level. Likewise, in July 2020, Law 27.553 concerning electronic prescriptions, was approved. It expedited prescriptions of misoprostol to carry out abortions via telemedicine. It removed the prerequisite of in-person visits to a health center to get the physical prescription. Colombia has been working since 2006 on removing bureaucratic obstacles and administrative barriers in access to the practice by rulings from the Constitutional Court (19). During the pandemic, previously approved strategies were put into practice and streamlined to implement the provision of abortions through telemedicine and other mechanisms for remote assistance or professional consultation to get to remote areas. However, accessibility was much better in private and specialized healthcare centers with a long tradition in sexual and reproductive care than in the public and social security systems.

All countries imposed biosafety measures to reduce the risk of infection, but some did not provide the necessary materials. In those cases, accessibility was affected. These measures led to the exclusion of low-income end users lacking the resources needed for an abortion. This happened in most of the monitored countries, given that most did not cover the costs of biosafety equipment such as face masks and gloves or the PCR tests required to enter a health center. For example, at the time we conducted our research, PCR tests were required to enter all healthcare facilities in Bolivia, costing about 70 American dollars (22% of the minimum monthly wage in the country at the time).

We also see wide disparities in the prerequisites for accessing abortion services in each country before the pandemic, and how these were either eased or tightened during the crisis (see Table 7). Countries such as Uruguay impose conditions that are not clinically necessary for safe abortion, such as a “reflection time,” which is a five-day waiting period between the initial appointment and the abortion service. Another requirement for requesting a legal abortion in Uruguay is to have been a resident in the country for a minimum of 1 year. These conditions were upheld in Uruguay despite the mobility restrictions caused by the pandemic. Likewise, in Ecuador and Peru hospitalization requirements and medical boards (responsible for approving abortion requests) remained in place in second-level healthcare services, even for early abortion.

**Table 7:** Prerequisites for access to abortion previous to the pandemic.

**Country**	**Prerequisites for access to abortion**				
	**Waiting period/more than 1 Appt**.	**Hospitalization/2 level or higher**	**Archived prescription**	**Rape report**	**Residence in country**
Argentina			X		
Bolivia		X			
Brazil		X			
Chile		X			
Colombia					
Ecuador	X	X	X		
Peru	X	X	X		
Salvador					
Uruguay	X				X

*Source. Prepared by authors with data from the “Reproductive Health is Vital” monitoring*.

*The / stands for partially*.

Requirements for abortion services because stricter during the pandemic in most of the countries studied, as seen in Table 8. Most countries kept the same requirements as those enforced before the pandemic, and only some enabled the use of technology for remote assistance for abortions. Ecuador, Peru, Chile, and Bolivia, for example, maintained the requirement of hospitalization in a second-level (or higher) healthcare center, overlooking the WHO's recommendation to guarantee access to safe abortion at all levels of care and on an outpatient basis for the first 12 weeks (20).

**Table 8:** Conditions for access and provision of abortion services during the first year of the COVID-19 pandemic.

**Country**	**Conditions for access and provision of abortion during the pandemic**								
	**Telehealth**	**Electronic prescription**	**Outpatient abortion**	**Medication without in-person visit **	**Rape report**	**Waiting period/more than 1 Appt.**	**Residence in Country**	**Archived prescription**	**Negative PCR**
Argentina	X	X	X	X					X
Bolivia									X
Brazil					X				X
Chile				X					X
Colombia	X	X	X						X
Ecuador						X		X	X
Peru						X		X	X
Salvador									
Uruguay			X			X	X		X

*Source. Prepared by authors with data from the “Reproductive Health is Vital” monitoring. The / stands for partially*.

Few countries eased the requirements, implemented flexible medical care circuits, and sought to strengthen policies to facilitate access during the pandemic. In Argentina, telehealth, electronic prescriptions, and the dissemination of information via the 0800 sexual health hotline allowed the changes and regulatory adaptations to better translate into real access to safe abortion services for users, for example.

Thus, we can see how the regulatory framework that guarantees access to safe abortion is different for each of the nine countries in this study. Furthermore, all the regulatory modifications made as a result of the pandemic could have lasting effects over time. In countries that increased access, there could be a positive impact. By contrast, in countries that created obstacles to access, without any reasonable justification, restrictions could be tightened.

### Access to Safe Abortion During the Pandemic

In the countries that provided data on the number of legal abortions carried out during the pandemic there was a significant reduction in the provision of both surgical and medical abortions compared to 2019, except for Brazil, which we will mention further on (see Table 9).

**Table 9:** Variation in number of legal abortions performed during 2019–2020.

Country*	**Period of 2019 and 2020 compared**	**Abortion variation 2019–2020 %**	**Source**
Brazil	January–June	+10.8	Instituto Nacional de Estadística - INE
Bolivia	January–July	−65	Instituto Nacional de Estadística - INE
Chile	January–June	−21	Instituto Nacional de Estadística - INE
Colombia	January–June	−19.6**	Departamento Administrativo Nacional de Estadística – DANE
Ecuador	March–July	−68.7	Ministerio Salud Pública - MSP
Peru	January–Sept	−86	Ministerio Nacional de Salud - MINSA

*Source. Prepared by authors with data from the “Reproductive Health is Vital” monitoring*.

^*^Argentina and Uruguay did not provide this data. El Salvador does not have official data because abortion is totally banned.

^**^This data did not come from official sources; it was developed by the Center for Reproductive Rights for this study-CDR.

Peru, Ecuador, and Bolivia showed the highest drop in abortion services provided in public health systems, particularly Peru with a decrease of 86%. The consequences of this are: increased risk of unsafe practices, complications, deaths, and medium and long-term consequences due to unwanted pregnancies (21). In these countries, the increase in maternal mortality is a sign of the need to recover reproductive health service provision. In Peru, the total number of maternal deaths increased by 12%, and in Ecuador by 21% during the period studied (Table 10) (22).

**Table 10:** Variation in maternal mortality total numbers 2019–2020.

**Country**	**Period of 2019 and 2020 compared**	**Variation 2019–2020 %**	**Source**
Brazil	January–June	+7.2	Instituto Nacional de Estadística - INE
Chile	January–September	+39	Instituto Nacional de Estadística - INE
Ecuador	First 40 weeks of the year	+21.6	Ministerio Salud Pública MSP - epidemiological magazine
Peru	First 27 weeks of the year	+12	Encuesta Demográfica y de Salud Familiar - ENDES

*Source. Prepared by authors with data from the “Reproductive Health is Vital” monitoring. The table shows variation in total numbers of maternal dead during the same period of time of 2019 and 2020*.

Brazil was the only country that reported more legal abortions (Table 9). Even though, according to data collected by the Anis institute, 55% of the facilities authorized to carry out pregnancy terminations suspended this service during the pandemic, the total number of abortions was higher than the previous year. A hypothesis set out by local researchers to explain this increase is that the issuance of regulations about abortion made its legality visible to both citizens and healthcare services. Before the pandemic, the local perception, encouraged by policies that did not promote access to termination of pregnancy services, was dominated by ignorance and the assumption that abortion was completely illegal. This is similar to what happened when procedural regulations (protocols, guidelines, or directives) that boosted access to abortion were issued in Argentina (2007), Ecuador (2015), and Perú (2014) (23). In 2020, the national government of Brazil approved Ordinance 2282 Procedure for Justification and Authorization of Interruption of Pregnancy, within the scope of the Unified Health System-SUS (27th August). Although it imposed unjustified prerequisites to accessing abortion, such as a mandatory ultrasound scan forcing women to hear and see fetus heartbeats, and a police report in case of rape, it did get a lot of media coverage. The ordinance and the possibility of accessing this service caught the general public's attention, and it may be why there was an increase in the requests received by public healthcare services in the months that followed. The ultrasound requirement was overruled after demonstrations (on the streets, social media and before authorities) against the measure, especially given that many human rights organizations classified it as tortuous, cruel, inhuman, and degrading for pregnant women (24). However, the issue took hold in the media, and information about the right to have an abortion reached millions of people.

Finally, most of the countries monitored had the necessary supplies to carry out abortions both surgically and medically. However, only Argentina reported extraordinary purchases (emergency or last-minute purchases, exceeding the ordinary budget) considering the increase in the demand for this service given the lack of access to contraception, the rate of domestic and sexual violence during home confinements, postponed family projects because of the health and economic crisis, among others (25).

In Peru for instance, we discovered misleading and unsafe services that offered access to abortion and scammed women. In Peru the *Safe Abortion Information* hotline that provides safe abortion counseling received complaints about counterfeit medications and high costs. The number of calls received by this hotline increased by 400% compared to the previous year. Women reported web pages and private phone lines advertising at-home medical abortion services that actually provided misleading information and forged medication.

### Barriers to Accessing Abortion During the Pandemic

The COVID-19 pandemic led to a shortage in human, infrastructure, and financial resources in general and especially for reproductive healthcare. Even though abortion in any form is a simple and relatively inexpensive procedure, the lack of available supplies, facilities, and trained personnel became barriers that discouraged users and hampered access to abortion services, particularly during the first few months of the pandemic. In countries such as Peru and Ecuador, the almost total shutdown of primary healthcare during the first semester of 2020 affected the provision of contraceptives, increasing the risk of unwanted pregnancy and, therefore, the need for legal abortions. In Brazil, as previously mentioned, according to data gathered by Anis, 55% of the facilities that provided abortions before the pandemic suspended this service in 2020.

In addition to this, the mobility restrictions, among other strict lockdown measures, prevented many users from accessing abortion services. In addition, fear of infection prevented many people from going to healthcare centers for months. In Ecuador, a non-representative survey of healthcare service users carried out by Surkuna identified that one of the barriers to access was fear of infection. We saw similar results in a survey carried out by Miles in Chile.

Accessibility to abortion services varied widely within each country. In rural and peripheral or hard-to-access sites, which had experienced deficiencies in health coverage even before the pandemic, these gaps grew wider. Even though telemedicine helped reach areas that lacked access in Colombia and Argentina, there were still many logistical obstacles to providing abortions: delivering medication proved to be quite a conundrum considering the scarcity of healthcare personal, mobility restrictions and so on. Likewise, referring cases that required hospitalization, or treating girls or adolescents, required a great deal of logistical coordination on behalf of healthcare providers. It should also be noted that most countries did not use telehealth for abortion services (Table 8).

Furthermore, access to technology and additional costs incurred by the pandemic, which in many cases were not covered by the State or private health insurance companies or social security, became prohibitive and prevented many users from accessing services. In Colombia the Center of Reproductive Rights identified lack of access to digital platforms and the internet as a significant barrier in rural areas, meaning patients and health teams did always have access to the necessary technology to implement telehealth, for instance.

A challenge that was persistently reported by all monitored countries was the lack of adequate and timely information about abortion care pathways. This meant that, in many cases, women had to travel to more than one healthcare center to find a provider, and were exposed to hostile contexts and ill-treatment. Ecuador, Colombia, and Peru reported such cases, with indigenous and migrant women in particular experiencing violence when asking for an abortion. This was the case for a Venezuelan woman we interviewed who lived in Ecuador:

“*I had a miscarriage because I could not get any medical attention. Then I went to the hospital to get a cleaning. I got yelled at when I was at the healthcare center. […] I think they treat us poorly because of our nationality. You feel discriminated against, you feel bad, and you start thinking that it's because you're Venezuelan; honestly, I don't know why they treated me like that.”*

The scarcity of supplies in healthcare services led to discriminatory measures that affected vulnerable groups such as Venezuelan migrants in all the countries in the region, especially Ecuador, Colombia, and Peru. As in the emblematic case of *Diana Alemán* in Peru, many health systems rejected migrants and, in many cases, their pregnancy termination requests were dismissed (26). *Diana Alemán*, a Venezuelan migrant, went to the María Auxiliadora Hospital on Saturday, July 4th, 2020, due to a miscarriage. When she was at the hospital, staff threatened to call immigration authorities alleging she had had an illegal abortion. Diana was hospitalized and found dead that same night. According to the hospital, she jumped out of a window on the third floor. Her family does not believe it was suicide. Fear of penal consequences and social stigmatization can lead to suicide, as proven by studies that link the inaccessibility of abortion to suicide. It is especially true in high-pressure contexts, such as that of the COVID-19 pandemic (27).

### Access to Abortion During the Pandemic: Best Practices

The diversified strategies for providing care and disseminating information deployed by the different countries has the potential to produce positive results beyond the pandemic. Although we believe these efforts did not suffice in light of the drastic drop in access to abortion services (Table 8), it is paramount to highlight the best practices.

Establishing helplines that informed users about their rights, services, and healthcare delivery pathways was vital. When users used these hotlines to schedule telemedicine appointments to receive electronic prescriptions for medications and supplies, access to healthcare services improved substantially. Specialized private services in Colombia made these types of hotlines available. The *0800SaludSexua*l hotline in Argentina is a good example from the public system. The hotline specializes in sexual and reproductive health counseling services, referring users to healthcare professionals who can perform the intervention close to where they live. The hotline also receives complaints and grievances related to inaccessibility and has a team dedicated to following up on these issues in order to overcome these barriers and promote timely access to services. The Bolivian telehealth hotline is used for triage and scheduling appointments.

Prescribing abortion medication during the first appointment, including via telemedicine, led to positive results in terms of access to abortion and helped to avoid abortion-related complications.

The efforts of abortion health providers in Argentina and Colombia were noteworthy. They used social media such as WhatsApp, Telegram, and Messenger to make video calls, and scheduled appointments by text messaging patients who needed help with legal abortions. They also sent photos of the physical prescriptions to places that did not have the technology to access electronic prescriptions, and all of this was carried out via telehealth in order to avoid unnecessary contact with health professionals.

We must also highlight the important role that civil society played in advocating for ensuring the right to abortion in the region. The dissemination of information via counseling and helpline services, doulas (female companions for outpatient pregnancy termination treatments), free or low-cost private medical and legal consultations; the production of educational resources, and data collection to identify barriers and facilitators during the COVID crisis have been indispensable tools for ensuring timely access to healthcare services and for updating public policies and actions to improve health system responses. A clear example of this collaborative work was seen in the Hospital de Clínicas de Uberlandia. The Anis Institute worked alongside the Comprehensive Care Center for Victims of Sexual Violence linked to the Hospital de Clínicas da Universidad Federal de Uberlândia, in Minas Gerais (Brazil), to develop two key tools for abortion care. The first, a *Protocol for Legal Abortion via Telehealth*, which may not represent a change in State policy but is nevertheless an important measure and point of reference for safe abortions. This protocol led to the first legal abortion via telehealth; it was also the first time in three decades that using misoprostol was allowed outside of a hospital environment in the public health arena. The second key tool was a protocol for abortions in the second trimester. The document confirms the legality of abortion in certain situations and provides guidelines for its provision during this crisis.

The working and technical groups implemented by national authorities in Chile and Argentina are another example of the important role played by civil society. These groups were created to share relevant information regarding barriers and facilitators, learn from positive experiences, benefit from civil society's expertise, and to develop adequate policies to address reproductive health needs during the pandemic. In Chile, for example, the Ministry of Women and Gender Equity led these groups, in which the sexual and reproductive rights working group was able to share and analyze access to abortion information. In Argentina, the National Ministry for Sexual and Reproductive Health established weekly meetings with representatives from the country's provinces to share information. It also implemented periodic meetings with civil society, and a WhatsApp group to facilitate permanent communication concerning access to services, including abortion, during the pandemic.

## Discussion

The goals of this study are to analyze information about the provision of reproductive healthcare services in Latin America during the COVID-19 outbreak, to demand accountability, and to contribute to the dissemination of best practices and favorable innovations for legal and safe access to abortion services. For this purpose, we gathered the available evidence during 2020, working with civil society organizations in nine Latin-American countries: Argentina, Bolivia, Brazil, Chile, Colombia, Ecuador, El Salvador, Peru, and Uruguay.

Even though six of the nine countries declared reproductive health as essential on paper, the reality is that these declarations did not translate into necessary and steadfast actions, and all nine countries faced restrictions and barriers to accessing these services.

In line with other recent research (28), our study shows the negative impact of COVID-19 on reproductive healthcare services, including abortions—with a reduction of up to 86% in the total number of abortions in one of the countries studied. We also noted great disparity in governmental responses on the matter, with Argentina at one end of the spectrum, reforming its law and being flexible with requirements, and Brazil at the other end, where more women were reported for having clandestine abortions and the government refused to consider reproductive health as an essential service.

Maternal mortality is a strong indicator; it reveals the cost of restricting or not guaranteeing access to reproductive healthcare services. Our research revealed a trend between March and August of 2020 of rising maternal mortality ratio in the region compared to the previous year (22). Peru is possibly the country that most dramatically reflects this finding; by the time we finished our research for this article, 433 pregnant women had died (between January and December 2020), a death rate the country had not experienced for a decade. The maternal mortality ratio kept rising, increasing by 42% compared with the same period of the previous year (29). “Overnight, pregnant women were left without available healthcare services” and “we could have probably avoided these deaths if those services hadn't been neglected” because “97% of the direct and indirect causes could have been avoided,” stated the Dean of the Obstetric Association of Peru, Margarita Pérez in an interview with EFE magazine (30). Maternal mortality is, once again, a priority in the global agenda, driven by this critical situation and promoted by the SDGs.

The maternal mortality ratio (MMR) for Latin America in 2019 was 67.2 deaths for every 100,000 live births; during the pandemic, these numbers, already stagnant, variable and irregular, took a backslide, worsening MMR. According to the UNFPA, there are three scenarios: the more optimistic one, where the regional MMR would backslide 5 years, the neutral one, where this ratio could backslide 13 years; and the grim one, where it rolls back 20 years (31).

When the pandemic began, we had already had similar experiences (SARS, EBOLA, N1H1 influenza, and Zika in Latin America) in which the lack of an efficient response in the provision of essential healthcare services, whether or not they were related to the outbreak, had a significant impact on mortality and morbidity rates (6, 32). These experiences suggested that States should adopt concrete measures to secure adequate and timely provision of essential services in order to reduce rates. They should do this by guaranteeing supplies, personnel, and procedures adapted to the conditions of the crisis before healthcare services become overwhelmed. Furthermore, many global, regional, and local organizations also emphasized the need to protect certain reproductive healthcare services, such as prenatal care, abortion, and contraception, in the first few months of the pandemic (6, 33–39).

Be that as it may, the COVID-19 pandemic changed the preexisting global context for sexual and reproductive health in many ways (40–42). These changes may be temporary, or they could be here to stay. Telehealth, providing abortions at the primary care level, outpatient abortions (or home abortions), electronic prescription of misoprostol are initiatives that could become more widespread and improve the quality of access to legal and safe abortion. On the other hand, undesirable situations, such as delays due to access restrictions, lack of available abortion care professionals, constraints for home abortions, among others, might persist after the COVID crisis is over.

There definitely seems to be strong resistance to changing standards and prerequisites to make legal and safe abortion services more accessible (Interestingly, there is not as much resistance in places with more legal frameworks, especially regarding abortion regulation models in Argentina or the court decisions recognizing the right to abortion in Mexico, Ecuador, Colombia, and the Inter-American Court for El Salvador). In fact, many of the legal requirements have no clinical justification: waiting periods, legal residency for a given period, or hospitalization for early abortion have clearly shown the detrimental effects on the user's health and rights. Having to go to more than one appointment during mobility restrictions entailed unnecessary extra efforts for users, higher risk of infections, and inefficient use of human resources, among others.

Only Argentina and Colombia officially adopted telehealth for the provision of legal abortion. These two countries were the only ones to carry out abortions at the primary care level before the pandemic. Only one of the nine countries studied was flexible concerning care prerequisites, which suggests that the best countries at ensuring the availability and accessibility of abortion services were the ones that had already made significant strides in this area. In Argentina, for example, a nationwide broad social and legislative debate on abortion was already underway, and a national health policy including abortion care (on justified grounds) had been formulated over a period of at least 10 years (43), albeit it gradually and with irregularities.

Resistance to abortion is not just found in Latin America. Restrictions have been imposed in other parts of the world too, and we see similar reluctance on behalf of governments to modify prerequisites and safe abortion provision models. In Europe, according to Moreau et al. research, abortion care was available during the pandemic to varying extents in 39 countries, banned for non-medical reasons in six countries (Andorra, Liechtenstein, Malta, Monaco, San Marino, and Poland) and suspended in Hungary due to a ban on non-life-threatening surgeries in state hospitals (44). In Northern Ireland, the country with the most restrictive laws until 2019, the Abortion Guidelines came into effect on March 31st, 2020, just after the UK lockdown, and did not allow—unlike the rest of the UK and Ireland—home use of mifepristone (45). Despite several recommendations and pressure from family planning clinics, NGOs, professional associations, including the Royal College of Obstetricians & Gynecologists and the Faculty of Sexual & Reproductive Healthcare, this has not changed, showing a long-lasting resistance to making abortion easier (45). Along these lines, and according to Moreau et al.'s research, medical abortion care during COVID-19 was observed in 13 of the 39 European countries surveyed, and only six implemented official policy amendments. These changes were considered temporary and pertained to the expansion of home-based medical abortion and methods for dispensing mifepristone. Telemedicine for medical abortions, previously only allowed in some regions of Sweden before COVID-19, became legal in five additional countries during the pandemic: England, Wales, Scotland, France, and Ireland. However, none of the countries expanded the legal gestational age limit for abortions, and none of the 12 surveyed countries that require mandatory waiting periods officially changed this regulation (44).

In the United States, we saw a wave of conservative regulations and other restrictive local measures that gained traction during the pandemic in several States of the country (46). Admittedly, even though the United States Supreme Court ruled in 1973 that abortion was a constitutional right until the fetus became “viable” (interpreted to be at around 24 weeks), several States approved laws forbidding abortion at earlier gestational ages. The law in Texas allows anyone to report somebody trying to help a person get an abortion after 6 weeks, and the law in Mississippi outlaws abortions after 15 weeks. In 2022 the Supreme Court will analyze these two laws, and there is a possibility that the Court could overturn the Roe ruling and restrict the right to have an abortion or decide that each State can establish its own abortion laws. It opens the doors to local regulations that, in practice, will make abortion services inaccessible. At any rate, this could mean that abortion could become extremely hard to get in at least 26 of the 50 States in the United States, marking the growing conservative radicalization of the country (47). We already see some of this happening: the pandemic has caused several disruptions to abortion service availability, including clinic closures (48) driven by unreasonable governmental requirements or bans specifically targeting abortion services (e.g., forbidding the use of telemedicine for home abortions in some States) giving rise to what we now call “the politics of abortion exceptionalism” (49).

As our findings suggest, the pandemic became a catalyst for what was already happening in each country. Brazil and Argentina represent, respectively, two case studies at opposite ends of the spectrum. Brazil hardened its policy on access to abortion, while in Argentina, the COVID-19 crisis did not worsen but rather favored reopening the legislative debate, resulting in a new law on legal pregnancy termination. Yet all that has happened and most probably will happen in Latin America is connected with the so-called green wave, a new movement that has reached even the most conservative countries. The movement persisted during the COVID-19 outbreak. It is a broad social movement in favor of legal and safe abortion, with high visibility, diversity, and the notable participation of young women. It shows openness toward public debate, resonates with feminist arguments and language, and has been well-received by decision-making institutions (50, 51). Just as the 1970s was the decade in which laws and rulings legalized abortion in most of Europe and the United States—the global north, the first few decades of the twenty-first century might be remembered as the green wave era in Latin America.

## Strengths and Limitations

Information gathering during the first few months of the pandemic in 2020 proved to be a strength and a limitation of the study.

We developed indicators based on several prior studies and tailored them to the objectives and feasibility of this study. The findings from this research enabled us to draft nine national reports and one regional one. We published some of the results on digital media to disseminate our findings and share the best practices implemented by certain countries in the region.

This research was possible thanks to high levels of buy-in and engagement across civil society in all nine counties, the regional coordination of CLACAI, and donor support.

Despite the strengths mentioned above, there are three limitations that cannot/must not be overlooked. First, the short time frame for the research: March–September 2020. Second, we were not able to access complete statistical data for the nine countries (given that the study ended in October 2020), as in many cases this was not publicly available till the following year. Third, we were not able to obtain data for all the indicators in all nine countries.

Finally, we must mention that in Latin America there are significant gaps in data production and the timely disclosure of health statistics used to create evidence-based public policies (52). Although the region was one of the fastest to approve constitutional and legal transparency regulations, the production of timely data, especially in health matters, is still deficient (53), making research and analysis of health outcomes in real-time especially challenging.

## Conclusions

We found that there are difficulties in terms of availability and accessibility of abortion health services in Latin America, confirming the negative impact of COVID-19 in this regard. We would like to make two observations. First, significant differences were identified between the nine countries examined in this study, which can be partially explained by the discrepancies in each country's specific political situation and abortion policies. Second, our research not only revealed restrictions and barriers to accessing abortion, but also found that innovative solutions such as telehealth had been implemented by some countries for the first time as a result of the pandemic. These innovations could lead to positive outcomes by making abortion care more widely available once the health crisis is over.

Given the possibility of lowering the maternal mortality ratio by offering access to safe abortion services, countries should include abortion at all levels of care and also increase and expand the use of telehealth at the primary care level, thereby helping to ensure the availability of medication (misoprostol and mifepristone). Some of the proposed initiatives are already being adopted in a few countries in the region, as outlined in this paper, and these countries could become good examples for other countries, helping them to transition from temporary responses to the COVID-19 crisis to developing quality standards in regulations and everyday health practice. These interventions are a necessary response to some of the green wave's demands and will help to tangibly and permanently promote the rights of thousands of women, girls, and teenagers across Latin America.

## Data Availability Statement

The original contributions presented in the study are included in the article/Supplementary Material, further inquiries can be directed to the corresponding authors.

## Ethics Statement

Ethical review and approval was not required for the study on human participants, in accordance with the local legislation and institutional requirements.

## Author Contributions

AR, SA, and SC: conception of the study and manuscript writing. SA and AR: study design, data collection, organization of the database, and data analysis. SC: critical revision of the study design and revision of the data analysis. AR: lead on the drafting of the manuscript. All authors contributed to manuscript revision, read, and approved the submitted version.

## Funding

This work was supported by New Venture Fund (NVF), Wellspring, IPPF -SAAF and Guttmacher Institute.

## Conflict of Interest

The authors declare that the research was conducted in the absence of any commercial or financial relationships that could be construed as a potential conflict of interest.

## Publisher's Note

All claims expressed in this article are solely those of the authors and do not necessarily represent those of their affiliated organizations, or those of the publisher, the editors and the reviewers. Any product that may be evaluated in this article, or claim that may be made by its manufacturer, is not guaranteed or endorsed by the publisher.
